# Perceptions of Artificial Intelligence Among Gastroenterologists in Italy: A National Survey

**DOI:** 10.3390/cancers17081353

**Published:** 2025-04-17

**Authors:** Marcello Maida, Sandro Sferrazza, Giulio Calabrese, Giovanni Marasco, Alessandro Vitello, Manuele Furnari, Ivo Boskoski, Emanuele Sinagra, Antonio Facciorusso

**Affiliations:** 1 Department of Medicine and Surgery, University of Enna ‘Kore’, 94100 Enna, Italy; 2 Gastroenterology Unit, Umberto I Hospital, 94100 Enna, Italy; 3 Gastroenterology and Endoscopy Unit, “ARNAS Civico-Di Cristina-Benfratelli” Hospital, 90127 Palermo, Italy; 4 Department of Medical and Surgical Sciences, University of Bologna, 40126 Bologna, Italy; 5 IRCCS Azienda Ospedaliera Universitaria di Bologna, 40138 Bologna, Italy; 6 Division of Gastroenterology, Department of Internal Medicine, University of Genoa, 16126 Genoa, Italy; 7 IRCCS Ospedale Policlinico San Martino, 16132 Genoa, Italy; 8 Digestive Endoscopy Unit, Fondazione Policlinico Universitario Agostino Gemelli IRCCS, 00136 Rome, Italy; 9 Gastroenterology Unit, Fondazione Istituto San Raffaele Giglio, 90015 Cefalù, Italy; 10 Gastroenterology Unit, Department of Experimental Medicine, University of Salento, 73100 Lecce, Italy; 11 Clinical Effectiveness Research Group, University of Oslo, 0313 Oslo, Norway

**Keywords:** artificial intelligence, gastroenterology, endoscopy, survey, Italy

## Abstract

**Background**: An extensive body of evidence regarding artificial intelligence (AI) in gastroenterology is currently available, but the transition of these technologies to the bedside is still in progress. This national survey aims to assess the perceptions of AI among gastroenterologists in Italy. **Methods**: A total of 320 Italian gastroenterologists and trainees in gastroenterology were invited to answer a web-based survey. A sub-group analysis between the two categories was performed. **Results**: Data from 150 respondents were analyzed. Of these, 67.3% were gastroenterologists and 32.7% trainees. Notably, 99.3% reported familiarity with AI in gastroenterology, with 49.3% currently using AI tools, indicating high levels of awareness and current use. Participants expressed low concerns regarding reliability on a 0-to-10-point scale (median = 4), legal and ethical issues (median = 5), and data protection (median 5), whereas regulatory concerns were moderate (median = 6), representing a key concern. Subgroup analysis revealed that male gastroenterologists had a higher understanding of AI (median = 7) compared to females (median 6, *p* = 0.036). Additionally, older gastroenterologists showed greater ease of use in AI endoscopy tools (median 8 vs. 7, *p* = 0.040) and raised more regulatory concerns (median 7 vs. 6, *p* = 0.036). **Conclusions**: The data from this survey show that Italian gastroenterologists have high-level awareness and a favorable perception of AI systems, with a good diffusion of AI tools across the national territory and reasonable concerns about regulatory issues, raising attention on international AI regulations.

## Simple Summary

Artificial intelligence (AI) is becoming increasingly relevant in gastroenterology, with new technologies designed to assist in detecting and managing digestive diseases. While AI can potentially improve diagnostic accuracy and efficiency, its integration into clinical practice depends on how healthcare professionals perceive it. This study explores the opinions and experiences of gastroenterologists across Italy regarding AI in their field. By gathering insights through a national survey, we aim to understand the level of awareness, current usage, key concerns, and socio-demographic differences related to AI adoption. Our findings indicate a generally high level of awareness and positive attitude toward AI, with significant interest and primary usage in its role in endoscopy and clinical decision-making. However, key challenges are represented by regulation and implementation issues. This research provides valuable insights that can guide future policies, training programs, and the development of AI tools that align with the needs of gastroenterologists.

## 1. Introduction

The wave of artificial intelligence (AI) is sweeping all fields of medicine, including gastroenterology. In recent years, AI has significantly transformed the healthcare landscape, offering innovative solutions that enhance diagnostic accuracy, optimize treatment plans, and streamline healthcare delivery. The incorporation of AI technologies has the potential to reduce human biases, provide diagnostic support, improve clinical decision-making, and standardize outcomes assessment in scientific research [1]. This burgeoning technology has enabled the analysis of vast datasets, facilitating personalized medicine approaches and improving patient outcomes across various specialties. A scoping review of randomized controlled trials (RCTs) on AI in clinical practice shows a growing interest in AI across various clinical specialties and locations, with gastroenterology playing a significant role by representing 37 of the 86 examined RCTs (43%) [2].

Among all AI tools recently introduced in gastroenterology, computer-aided polyp detection (CADe) is the most promising. This system has been successfully implemented in colonoscopy to increase sensitivity in identifying colon lesions and support human diagnostic performance [3]. Data from the literature show that CADe outperforms white light colonoscopy (WLC) in terms of increased adenoma detection rate (ADR) [4]. Nevertheless, this benefit has not been confirmed in the setting of FIT-based screening [5] or in real life under less controlled conditions [6]. Similarly, computer-aided polyp detection (CADx) has been developed to help endoscopists detect and manage colorectal lesions [4]. As a consequence, the benefits and cost-effectiveness of CADe and CADx for colorectal cancer (CRC) prevention are still under investigation. Other tools used in gastroenterology are AI-assisted chatbots such as ChatGPT (Chat Generative Pretrained Transformer, OpenAI Foundation, Mar 14 Version 2023). These are emerging as revolutionary tools that have the potential to provide educational support to patients through an accessible question–answer system [7]. As it has the potential to serve as a complementary resource for healthcare, it is currently being evaluated in various areas of gastroenterology, including nonalcoholic fatty liver disease [8], acute pancreatitis [9], *Helicobacter pylori* infection [10], and colonoscopy [11].

Despite the utility of this new technology, most of which are already available, and others under investigation, physicians’ perceptions and concerns about the current and future impact of AI in gastroenterology are not fully clear. Understanding physicians’ perceptions is crucial for the effective clinical adoption of AI, as acceptance and trust in these technologies significantly influence their integration into routine practice. A 16-question survey of 374 gastroenterologists by the American Society for Gastrointestinal Endoscopy AI Task Force showed an overall positive perception of using AI in clinical practice [12]. However, it raised concerns regarding its technical aspects and coverage of costs associated with implementation. Nevertheless, these data have been produced mainly in North America and may differ by country due to different cultural aspects and local resource availability.

A similar finding was present in a web-based survey of 165 clinicians in the Asia–Pacific region: a generally favorable attitude toward adopting AI in gastroenterology practice was found, with participants expressing a strong intention to use AI tools when available [13]. However, the study also highlighted variations in acceptance, trust, and perceived risk, influenced by factors such as prior exposure to AI and individual perceptions of its reliability.

Moreover, the reproducibility of the data on fellows is hindered by variations in training processes across different regions [14] and data regarding Italy currently lacking. This national survey aims to explore the current use and perceptions of AI among gastroenterologists in Italy at a country level.

## 2. Materials and Methods

### 2.1. Study Design and Development of the Survey Questionnaire

This web-based cross-sectional survey was performed following the recommendations of the Consensus-Based Checklist for Reporting of Survey Studies (CROSS) [15] (File S1).

A 40-point multiple-choice questionnaire was designed by the working group during videoconference meetings with the following objectives:

- To identify the level of awareness of gastroenterologists regarding AI
- To identify the level of usage of AI by gastroenterologists
- To identify the concerns of gastroenterologists regarding the usage of AI
- To identify differences by socio-demographic variables.

The survey was structured as follows: (1) general information and demographic data, (2) awareness and familiarity with AI in gastroenterology, (3) current usage and perception of AI tools, (4) barriers and concerns, (5) training and education, and (6) future outlook.

### 2.2. Distribution of Questionnaire and Collection of Data

After approval by all components of the working group, the final version of the questionnaire was viewed via Google Forms. The link to access the survey was sent via email, together with a brief explanation of the project (the full version of the questionnaire is available as File S2), to 320 gastroenterologists and trainees in gastroenterology from all 20 regions of Italy. A first invitation was sent to all members, followed by two subsequent fortnightly reminders. Only responses from board-certified gastroenterologists were accepted. When possible, data from at least one physician from each regional hospital were collected to obtain a comprehensive picture of the national territory.

Ethical committee approval was not required for this type of survey due to the absence of patient data. All subjects agreed to participate voluntarily in the interview through informed consent for the collection, handling, and storage of data, which was included in the presentation of the questionnaire. Data collection took place from May to June 2024.

### 2.3. Statistical Analysis

The data were analyzed in aggregate form without including any personal identifying information. Continuous variables were reported as mean ± standard deviation (SD) or median and interquartile range (IQR), and categoric variables were summarized as frequency and percentage. Comparisons of variables were made by the Mann–Whitney, Kruskal–Wallis, Chi square test, and Fisher test as appropriate. A *p*-value of less than 0.05 was considered to indicate statistical significance. Subgroup analyses by gender, age, and working status were conducted. All statistical analyses were performed using SPSS v. 29.0 for Macintosh (SPSS Inc., Chicago, IL, USA).

## 3. Results

Our survey showed that Italian gastroenterologists have a generally positive perception of AI, with strong awareness and acceptance, particularly in endoscopy and clinical decision-making. Concerns about reliability, ethics, and data protection were low, while regulatory issues remained a moderate concern. Most participants were from academic hospitals, and AI use beyond endoscopy was limited (15.3%). These findings highlight both the growing interest in AI and the need for clearer regulations and broader adoption across different healthcare settings.

### 3.1. General Information and Demographic Data

Overall, 150 of the 320 invited physicians responded to the survey, resulting in a response rate of 46.9%. Data from all respondents were included in the analysis. The main characteristics of the participants in the survey are summarized in Table 1.

Overall, 65.5% were gastroenterologists and 34.5% gastroenterology trainees. In total, 55% were males, and the mean age was 37.0 ± 9.8 years. The median year of practice in gastroenterology was 6 (3–13). Half of the participants (54.7%) worked in academic hospitals, while the others in non-academic public hospitals (37.3%) or private hospitals (8.0%). The participants covered all 20 Italian regions. In detail, 42.7% were from Northern Italy, 17.3% were from Central Italy, and 40.0% were from Southern Italy (Supplementary Figure S1).

### 3.2. Awareness, Current Usage, and Perception of AI Tools in Gastroenterology

Almost all responders (99.3%) had ever heard about AI in gastroenterology. The median knowledge about AI tools in gastroenterology was 6 (5–8) on a scale from 1 to 10.

**Figure 1:**
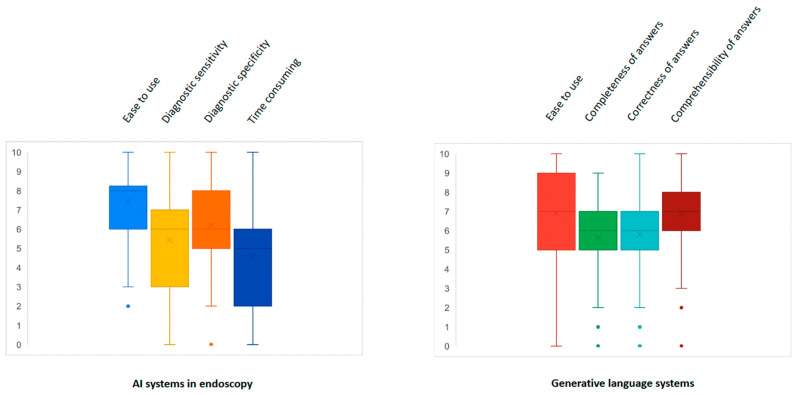
Perception of AI in endoscopy systems and generative language systems.

In real life, about one-half (49.3%) of participants used AI tools in endoscopy at the time of the survey. The median perception of AI systems for endoscopy was 8 (6–8) for simplicity in use, 6 (3–7) for diagnostic sensitivity, 6 (5–8) for diagnostic specificity, and 5 (2–6) for extension of procedure time on a scale from 1 to 10 (Figure 1). On the other hand, 40% of respondents also used generative language systems. In this regard, median perception was 7 (5–9) for simplicity in use, 6 (5–7) for completeness and for correctness of answers, and 7 (6–8) for comprehensibility of answers (Figure 1). Only 23/150 (15.3%) respondents used AI tools other than endoscopy ones and generative language systems, mainly in the hepatology and IBD settings.

### 3.3. Key Concerns and Barriers

Responders’ opinions on the main barriers to the spread of AI in gastroenterology in real life are costs (52.0%), difficulties in supply by hospitals (50.0%), doctors’ lack of knowledge or awareness of available AI technologies (50.0%), and the absence of guidelines on their use (56.0%).

**Figure 2:**
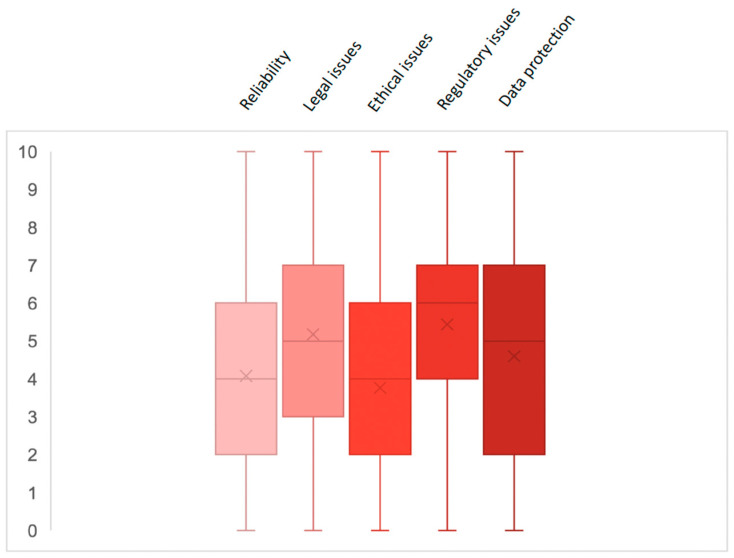
Concerns about using AI systems in gastroenterology.

About one-half of the participants raised a fair concern about using AI systems in gastroenterology. The main concern was on regulatory issues, with a median score of 6 (4–7) on a scale from 1 to 10, followed by legal and data protection, with a median score of 5 (3–7) and 5 (2–7), respectively (Figure 2). On the contrary, a fair concern was expressed regarding the reliability of the tools and ethical issues, with a median score of 4 (2–6) (Figure 2).

### 3.4. Training and Education

Overall, 79.3% of respondents believe that AI should be applied in the training of young gastroenterologists. The most appropriate modalities to train young gastroenterologists on AI were practice courses in person (80.7%), hands-on courses (64.7%), and online courses (32.7%).

### 3.5. Future Outlook

The majority of survey participants (91.3%) believe that AI will have a positive impact on endoscopic practice in the future and are optimistic regarding its potential to improve endoscopic procedures (93.3%).

Overall, 80.7% of respondents think that AI will be easily integrated into clinical practice (80.7%) in a median of 5 (5–10) years.

All responses are summarized in Table 2.

### 3.6. Subgroup Analyses by Socio-Demographic Differences

The subgroup analysis by gender of the survey participants showed differences in terms of knowledge about AI in Gastroenterology: 7 (6–8) vs. 6 (5–7) *p* = 0.036 for male and female, respectively. No other significant differences emerged in the subgroup analysis by gender, confirming a similar perception of AI between men and women (Table 3).

In the subgroup analysis by age groups (<40 years and ≥40 years), older gastroenterologists declared a higher knowledge about AI in Gastroenterology: 7 (6–8) vs. 6 (5–7) *p* = 0.005, respectively. Concerning the perception of AI systems in endoscopy, older gastroenterologists declared a higher simplicity in their use: 8 (7–9) vs. 7 (6–8) *p* = 0.040, respectively. Similarly, they perceived a higher correctness of responses produced by generative language systems compared to younger colleagues: 7 (5–8) vs. 5 (5–7), respectively, *p* = 0.013. Moreover, more experienced physicians showed significantly higher concerns about regulatory issues in using AI systems in gastroenterology: 7 (4–8) vs. 6 (4–7), respectively, *p* = 0.036 (Table 3).

Analyzing data by working status, practicing gastroenterologists declared a higher knowledge about AI in gastroenterology compared to trainees: 7 (6–8) vs. 6 (4–7) *p* < 0.001, respectively.

The assessment of perception in AI tools was comparable between practicing gastroenterologists and trainees except for simplicity in the use of AI systems in endoscopy: 8 (7–9) vs. 7 (6–8) *p* = 0.003, respectively.

No statistically significant difference was found in concerns about using AI systems in gastroenterology or in other items (Table 3).

No differences were found when comparing academic and non-academic centers.

## 4. Discussion

This large web-based national survey provides a comprehensive picture of the perception of AI tools among gastroenterologists in Italy.

First of all, we highlighted a proper awareness of AI in gastroenterology, with sufficient knowledge of current AI systems. Of note, perceived knowledge of AI in gastroenterology was significantly higher in men and in more experienced physicians (gastroenterologists versus trainees and in those aged >40 years versus <40 years).

In contrast to the 6.7% of reported AI use in clinical practice according to an American Society for Gastrointestinal Endoscopy AI Task Force survey [12], approximately half of the respondents in our study indicated they currently utilize AI.

This mainly concerns the use of tools introduced in the endoscopic setting, specifically CADe and CADx. This trend may suggest a heightened interest in diagnostic systems within endoscopy, but it could also be attributed to the earlier introduction of these systems compared to others.

AI tools were perceived as easy to use, with good confidence in their diagnostic performance. In this regard, they were perceived as easier to use among specialists and more experienced physicians. This was unexpected as younger physicians were assumed to be more familiar and comfortable with AI; however, it is important to note that the doctors over 40 were relatively young, with a median age of 49.5 years.

Similarly, another US survey found that 86% of physicians were interested in AI-assisted colonoscopy, and 84.7% believed that CADe would enhance their endoscopic performance [16].

In our study, a good diffusion of large language systems was recorded in approximately 40% of respondents, with greater application in the scientific field compared to the clinical one. These chatbots were considered easy to use, with sufficient performance for the completeness of the answers generated and good comprehensibility. The correctness of the answers was judged significantly higher by physicians ≥ 40. The latter have more significant experience in gastroenterology and, probably, greater reliability in judging the correctness of the information generated by the chatbot.

Approximately half of the respondents emphasized issues related to hospital costs and supply as barriers to the diffusion of AI. This is important because it highlights the need to allocate additional funds to healthcare facilities for technological updates, specifically AI. These findings are in line with previous studies, which found that the main concern regarding AI usage was its cost, cited by 75.2% of respondents [16,17].

In our study, half of the participants mentioned that the lack of awareness among doctors and the absence of specific guidelines might be potential obstacles.

This is consistent with a prior UK survey that indicated the greatest barrier to integrating AI into standard clinical practice was the lack of guidelines, reported by 92% of respondents [18]. To address this gap, it is important to focus on educating gastroenterologists and for scientific societies to develop and release specific guidelines.

Overall, participants expressed minimal concerns regarding reliability, legal and ethical issues, and data protection. However, they raised important questions regarding regulatory matters, which is perceived as an impacting topic also specifically in the field of endoscopy [19]. As expected, physicians over 40 years old demonstrated more significant concerns about these regulatory issues. This may be attributed to their cautious approach towards new technologies or their heightened awareness of regulatory matters due to their extensive professional experience. Moreover, according to this survey, respondents believe that AI will be integrated into clinical practice within a median of 5 years.

Finally, in line with the findings from the American Society for Gastrointestinal Endoscopy AI Task Force survey [10], over 90% of the respondents believe that AI will have a positive impact on endoscopic practice, with the potential to improve endoscopic procedures in the future. However, the perceived utility of AI in more complex aspects of clinical practice remains limited. In the aforementioned Asia–Pacific survey [13], clinicians placed less trust in CAD intervention compared to CADe and CADx. This highlights the varying levels of trust depending on the specific application of AI.

Another debated topic concerns the application of AI systems in trainee education. In this regard, about 80% of the respondents support the idea of incorporating AI technologies into the training of young gastroenterologists. This opinion is shared by both trainees and experienced gastroenterologists, indicating a widespread belief in the value of integrating AI into educational paths. This perception is supported by studies showing the positive impact of AI on endoscopist quality training [20,21,22].

Nevertheless, Italian medical training focuses on hands-on experience and clinical reasoning, which may limit the attitude of young physicians towards AI in our sample. Furthermore, cultural factors, such as the traditionally structure of Italian healthcare, may impact AI acceptance, as physicians might be more reluctant to depend on automated systems for decision-making.

Our study has several strengths. It is the first report on the perception of AI among gastroenterologists in Italy at a country level.

In addition, it has been conducted among both board-certified gastroenterologists and trainees. Furthermore, we achieved consistent participation from across the entire country. On the other hand, we must also acknowledge some limitations.

First, the small sample size, 54.7% from Academic Hospitals, affects the generalizability of the results.

Second, the study design did not ensure a systematic survey delivery to all Italian gastroenterologists, which may have introduced sampling bias and led to an overestimation of the results. As a result, it is likely that only the most interested or skilled gastroenterologists in AI have been selected.

Finally, only a small proportion (15.3%) of respondents reported using AI tools beyond endoscopy and generative language models. This suggests that AI integration in gastroenterology remains focused on specific applications, with limited adoption in areas such as pathology, predictive analytics, and administrative tasks. Larger studies comparing different populations in Europe are needed to explore the potential expansion of AI in these domains and implement guidelines and regulations.

## 5. Conclusions

The data from this survey show that Italian gastroenterologists have proper awareness and a favorable perception of AI systems, with a good diffusion of AI tools across the national territory, low concerns regarding reliability, legal and ethical issues, and data protection, but fair concerns about regulatory issues.

These findings further indicate the need to regulate the use of AI in clinical practice at a country level.

For instance, guidelines regulating how to use AI systems currently available should be released, so as to provide practical recommendations to specialists based on scientific evidence. Moreover, policies addressing ethical and legal issues should be released to make the use of AI systems among gastroenterologists easier and more confident at a country level. Further initiatives, beyond guidelines and policies on ethical and legal aspects of AI use, should be implemented to improve the acceptance and integration of AI in clinical practice.

In this regard, educational interventions are paramount for successful integration. Including AI modules in continuing medical education and residency programs will familiarize young gastroenterologists with these tools, facilitating their training. Practical workshops, online courses, and simulation-based training programs can support this educational need, allowing gastroenterologists to interact with AI tools in controlled environments. Additionally, fostering collaboration between AI developers and gastroenterologists will ensure that AI solutions are tailored to meet specific clinical needs, thereby enhancing their usability and effectiveness.

In this regard, it is mandatory to guarantee transparency between the manufacturer and user of the systems, displaying the data regarding its development, the training of the machine and its performance.

Finally, to enhance the training of young gastroenterologists, institutions should gradually introduce AI technologies. This approach aims to improve their familiarity with innovative tools, enhance their diagnostic and procedural skills, and prepare them to effectively integrate AI into clinical practice, ultimately optimizing patient outcomes.

In this effort, scientific societies can play a crucial role in promoting AI training across the country. Despite the relevance of these results, considering the methodological limitations of surveys, proper studies are needed to systematically assess these findings across the national territory.

## Figures and Tables

**Table 1:** Characteristics of the participants of the survey.

	N = 150
Age, years (mean ± SD)	38.3 ± 11.2
Gender N, (%)	
-Male	83 (55.3%)
-Female	67 (44.7%)
Working position N, (%)	
-Gastroenterologist	101 (67.3%)
-Trainee in Gastroenterology	49 (32.7%)
Year of practice in Gastroenterology (median, IQR)	6 (3–13)
Practice setting N, (%)	
-Academic	82 (54.7%)
-Public non-academic	56 (37.3%)
-Private	12 (8.0%)
Geographic areas N, (%)	
-Northern Italy	64 (42.7%)
-Central Italy	26 (17.3%)
-Southern Italy	60 (40.0%)

**Table 2:** Responses to the survey questions.

***Awareness and familiarity with AI in Gastroenterology***	
Have you ever heard of AI in Gastroenterology? N (%)	
-Yes	149 (99.3%)
How much is your knowledge about AI in Gastroenterology)	
1 to 10 (median, IQR)	6 (5–8)
***Current usage and perception of AI tools in Gastroenterology***	
Do you currently use AI tools in Endoscopy? N (%)	
-Yes	74 (49.3%)
Which AI endoscopic tool? * N (%)	
-Lesion detection (CADe)	39 (52.7%)
-Lesion detection and characterization (CADe/CADx)	43 (58.2%)
-AI in capsule endoscopy	17 (23.0%)
-AI in endoscopic assessment in IBD	1 (1.4%)
-AI in EUS	1 (1.4%)
What is your perception on AI systems in endoscopy? (median, IQR)	
-Ease to use (1 to 10)	8 (6–8)
-Diagnostic sensitivity (1 to 10)	6 (3–7)
-Diagnostic specificity (1 to 10)	6 (5–8)
-Extension of procedure times (1 to 10)	5 (2–6)
Do you currently use generative language systems? N (%)	
-Yes	60 (40.0%)
For what purpose? * N (%)	
-Clinical purposes	23 (35.9%)
-Informative purposes	20 (31.3%)
-Scientific purposes	38 (59.4)
-Other	5 (3.3%)
In your opinion, generative language systems can be useful: * N (%)	
-To help doctors in managing patients	63 (52.1%)
-To help patients acquire medical information	39 (32.2%)
-To help researchers in writing scientific articles	85 (70.2%)
What is your perception on generative language systems? (median, IQR)	
-Ease to use (1 to 10)	7 (5–9)
-Completeness of answers (1 to 10)	6 (5–7)
-Correctness of answers (1 to 10)	6 (5–7)
-Comprehensibility of answers (1 to 10)	7 (6–8)
Do you use AI systems in other areas of gastroenterology? N (%)	
-Yes	23 (15.3%)
In which setting? * N (%)	
-Hepatology	12 (50%)
-Pancreatology	7 (29.2%)
-IBD	12 (50%)
-Pathophysiology of digestive tract	4 (16.7%)
-Gastrointestinal oncology	5 (20.8%)
-Other	3 (12.6%)
***Barriers and concerns***	
What do you think are the main barriers limiting the spread of AI in gastroenterology? * N (%)	
-Costs	78 (52.0%)
-Difficulties in supply by hospitals	75 (50.0%)
-Lack of knowledge or awareness of doctors	75 (50.0%)
-Absence of guidelines on their use	84 (56.0%)
How concerned are you about using AI systems in gastroenterology? Years (median, IQR)	
-Reliability (1 to 10)	4 (2–6)
-Legal issues (1 to 10)	5 (3–7)
-Ethical issues (1 to 10)	4 (2–6)
-Regulatory issues (1 to 10)	6 (4–7)
-Data protection (1 to 10)	5 (2–7)
***Training and education***	
Do you think AI should be used in the training of young gastroenterologists? N (%)	
-Yes, I think it can facilitate and increase learning and training	119 (79.3%)
-No, I think it could represent a handicap in the training processes	24 (16.0%)
-I think its use is irrelevant for training purposes	7 (4.7%)
What do you think are the most appropriate modalities to train young gastroenterologists on AI? * N (%)	
-Clinical practice in the room	121 (80.7%)
-Hands-on courses	97 (64.7%)
-Online courses	49 (32.7%)
***Future outlook***	
How do you predict AI integration will impact endoscopic practice in the future? N (%)	
-Positive impact	137 (91.3%)
-Negative impact	2 (1.3%)
-Neutral impact	11 (7.3%)
Are you optimistic about the potential of AI to improve endoscopic procedures? N (%)	
-Yes	140 (93.3%)
Do you think AI will be easily integrated into clinical practice? N (%)	
-Yes	121 (80.7%)
In how many years do you think AI will be integrated into clinical practice? Years (median, IQR)	5 (5–10)

* Indicates that more than one answer was allowed.

**Table 3:** Subgroup analysis of responses by gender, age, and working position.

	Men (N = 83)	Women (N = 67)	*p*	Age < 40 (N = 100)	Age ≥ 40 (N = 50)	*p*	Gastroenterologists (N = 101)	Trainees (N = 49)	*p*
Have you ever heard of AI in Gastroenterology?									
-Yes	83 (100.0%)	66 (98.5%)	0.264	99 (99.0%)	50 (100.0%)	0.478	101 (100.0%)	48 (98.0%)	0.150
How much is your knowledge about AI in Gastroenterology?									
1 to 10 (median, IQR)	7 (6–8)	6 (5–7)	**0.036**	6 (5–7)	7 (6–8)	**0.005**	7 (6–8)	6 (4–7)	**<0.001**
Do you currently use AI tools in Endoscopy?									
-Yes	43 (51.8%)	31 (46.3%)	0.500	48 (48.0%)	26 (52.0%)	0.644	51 (50.5%)	23 (46.9%)	0.683
What is your perception on AI systems in endoscopy? (median, IQR)									
-Ease to use (1 to 10)	8 (7–9)	7 (6–8)	0.149	7 (6–8)	8 (7–9)	**0.040**	8 (7–9)	7 (6–8)	**0.003**
-Diagnostic sensitivity (1 to 10)	6 (3–7)	6 (4–7)	0.334	5.5 (4–7)	7 (3–8)	0.231	6 (3–7)	5 (4–7)	0.529
-Diagnostic specificity (1 to 10)	6 (5–8)	6 (5–7)	0.240	6 (5–8)	6 (5–7)	0.460	6 (5–8)	6 (5–8)	0.396
-Extension of procedure times (1 to 10)	5 (2–6)	5 (3–7)	0.060	5 (3–6)	5 (2–6)	0.847	5 (2–6)	5 (3–6)	0.960
Do you currently use generative language systems?									
-Yes	35 (42.2%)	25 (37.3%)	0.546	43 (43.0%)	17 (34.0%)	0.289	36 (35.6%)	24 (49.0%)	0.118
What is your perception on generative language systems? (median, IQR)									
-Ease to use (1 to 10)	7 (6–8)	6 (5–9)	0.301	7 (5–8)	8 (5–9)	0.682	7 (5–9)	7 (5–8)	0.661
-Completeness of answers (1 to 10)	6 (5–7)	5 (5–7)	0.773	6 (5–7)	6 (5–8)	0.059	6 (5–7)	6 (5–7)	0.665
-Correctness of answers (1 to 10)	6 (5–7)	6 (5–7)	0.553	5 (5–7)	7 (5–8)	**0.013**	6 (5–7)	6 (5–7)	0.403
-Comprehensibility of answers (1 to 10)	7 (6–8)	7 (5–8)	0.952	7 (6–8)	8 (6–8)	0.091	7 (6–8)	7 (6–8)	0.763
How concerned are you about using AI systems in gastroenterology in terms of:									
-Reliability (1 to 10)	4 (2–6)	4 (3–5)	0.759	4 (3–6)	4 (2–5)	0.301	4 (2–5)	5 (3–6)	0.063
-Legal issues (1 to 10)	5 (3–7)	5 (3–7)	0.842	5 (3–7)	7 (3–8)	0.064	6 (3–8)	5 (3–7)	0.305
-Ethical issues (1 to 10)	4 (2–6)	4 (1–6)	0.760	3 (1–5)	5 (2–7)	0.070	4 (2–7)	3 (0–5)	0.072
-Regulatory issues (1 to 10)	6 (5–7)	6 (3–7)	0.064	6 (4–7)	7 (4–8)	**0.036**	6 (4–7)	5 (5–7)	0.505
-Data protection (1 to 10)	5 (2–7)	5 (2–7)	0.584	5 (2–7)	5 (2–7)	0.520	5 (2–7)	5 (2–7)	0.488
Do you think AI should be used in the training of young gastroenterologists?									
-Yes, I think it can facilitate and increase learning and training	64 (77.1%)	55 (82.1%)	0.626	76 (76.0%)	43 (86.0%)	0.336	80 (79.2%)	39 (79.6%)	0.280
-No, I think it could represent a handicap in the training processes	14 (16.9%)	10 (14.9%)		19 (19.0%)	5 (10.0%)		18 (17.8%)	6 (12.2%)	
-I think its use is irrelevant for training purposes	5 (6.0%)	2 (3.0%)		5 (5.0%)	2 (4.0%)		3 (3.0%)	4 (8.2%)	
How do you predict AI integration will impact endoscopic practice in the future?									
-Positive impact	74 (89.2%)	63 (94.0%)	0.365	90 (90.0%)	47 (94.0%)	0.486	93 (92.1%)	44 (89.8%)	0.835
-Negative impact	2 (2.4%)	0 (0%)		1 (1.0%)	1 (2.0%)		1 (1.0%)	1 (2.0%)	
-Neutral impact	7 (8.4%)	4 (6.0%)		9 (9.0%)	2 (4.0%)		7 (6.9%)	4 (8.2%)	
Are you optimistic about the potential of AI to improve endoscopic procedures?									
-Yes	76 (91.6%)	64 (95.5%)	0.334	93 (93.0%)	47 (94.0%)	0.817	94 (93.1%)	46 (93.9%)	0.852
Do you think AI will be easily integrated into clinical practice?									
-Yes	70 (84.3%)	51 (76.1%)	0.205	77 (77.0%)	44 (88.0%)	0.108	81 (80.2%)	40 (81.6%)	0.835
In how many years do you think AI will be integrated into clinical practice? Years (median, IQR)	5 (5–8)	5 (5–10)	0.286	5 (5–10)	5 (5–8)	0.863	5 (5–10)	5 (5–10)	0.901

## Data Availability

Data are sharable upon reasonable request to the corresponding author.

## Supplementary Materials

The following supporting information can be downloaded at https://www.mdpi.com/article/10.3390/cancers17081353/s1, Figure S1. Map of participants (regions and cities). File S1, Table S1: Consensus-Based Checklist for Reporting of Survey Studies (CROSS) recommendation. Supplementary Table S2: Subgroup analysis of responses by geographical area in Italy. File S2: Full version of the questionnaire.
