# Supplementary material for: Perceptions of Artificial Intelligence Among Gastroenterologists in Italy: A National Survey
Source: Cancers (Basel). 2025 Apr 17;17(8):1353. doi: 10.3390/cancers17081353 (PMC12026144; doi:10.3390/cancers17081353)
Supplement: Supplementary file 1 [file cancers-17-01353-s001.zip › FIle S2.pdf]

Italian version

Perception of artificial intelligence among Gastroenterologists in Italy: a national survey.

Gentile collega, stiamo conducendo una survey nazionale con l'obiettivo di valutare la percezione e l'accettabilità delle metodiche di intelligenza artificiale tra i gastroenterologi in Italia.

Ti chiediamo di partecipare attraverso la compilazione di un questionario online che richiede circa 5 minuti del tuo tempo. Le risposte fornite verranno trattate in forma anonima e aggregata, unicamente per scopi scientifici.

~~* Indica una domanda obbligatoria~~

1. Proseguendo con questa intervista fornisco il mio consenso a salvare e utilizzare i dati forn per scopi scientifici e di ricerca.

   Contrassegna solo un ovale.

   - Acconsento
   - Non acconsento (fine della survey)

2. Sei un medico specialista o specializzando in Gastroenterologia? *

Contrassegna solo un ovale.

- Si
- No

SEZIONE 1 – Informazioni demografiche

3. Nome, Cognome *

4. Età *

5. Genere *

Contrassegna solo un ovale.

- Maschile
- Femminile
- Altro

6. Specializzazione *

Contrassegna solo un ovale.

- Specialista in Gastroenterologia
- Specializzando in Gastroenterologia

7. Anni di pratica in Gastroenterologia *

8. Nome Ospedale *

9. Tipo ospedale *

Contrassegna solo un ovale.

- Universitario
- Non universitario
- Privato

10. Città *

11. Area *

Contrassegna solo un ovale.

- Nord
- Centro
- Sud e isole

Sezione senza titolo

SEZIONE 2 – Consapevolezza e familiarità con l'AI

12. Hai sentito parlare di intelligenza artificiale in gastroenterologia? *

Contrassegna solo un ovale.

- Si
- No

13. Quanto conosci le applicazioni dell'intelligenza artificiale in gastroenterologia? *

Contrassegna solo un ovale.

	0	1	2	3	4	5	6	7	8	9	10	
Per	◯	◯	◯	◯	◯	◯	◯	◯	◯	◯	◯	Mi ritengo un esperto

SEZIONE 3 – Utilizzo dei sistemi di AI

14. Utilizzi attualmente sistemi di intelligenza artificiale in endoscopia? *

Contrassegna solo un ovale.

◯ Si
◯ No

15. Se si, quali?

Seleziona tutte le voci applicabili.

☐ Detezione (CADe)
☐ Detezione e caratterizzazione (CADe/CADx)
☐ Valutazione endoscopica delle IBD
☐ Sistemi di AI per videocapsula endoscopica
☐ Altro: ____________________

16. Quale è la tua percezione sui sistemi di intelligenza artificiale di endoscopia corrKentemente in commercio?

1) Semplicità di utilizzo

Contrassegna solo un ovale.

	0	1	2	3	4	5	6	7	8	9	10	
Estr	◯	◯	◯	◯	◯	◯	◯	◯	◯	◯	◯	Estremamente semplice

17. Quale è la tua percezione sui sistemi di intelligenza artificiale di endoscopia correntement in commercio?

2) Sensibilità diagnostica

Contrassegna solo un ovale.

	0	1	2	3	4	5	6	7	8	9	10	
Bas:	◯	◯	◯	◯	◯	◯	◯	◯	◯	◯	◯	Alto rischio di falsi negativi

18. Quale è la tua percezione sui sistemi di intelligenza artificiale di endoscopia correntement in commercio?

3) Specificità diagnostica

Contrassegna solo un ovale.

	0	1	2	3	4	5	6	7	8	9	10	
Bas:	◯	◯	◯	◯	◯	◯	◯	◯	◯	◯	◯	Alto rischio di falsi positivi

19. Quale è la tua percezione sui sistemi di intelligenza artificiale di endoscopia correntement in commercio?

4) Prolungamento nei tempi della procedura

Contrassegna solo un ovale.

	0	1	2	3	4	5	6	7	8	9	10	
Sca:	◯	◯	◯	◯	◯	◯	◯	◯	◯	◯	◯	Elevato impatto

20. Nella pratica medica hai mai utilizzato sistemi di linguaggio generativo (ad es. ChatGPT)?

Contrassegna solo un ovale.

- Si
- No

21. Se si, a che scopo?

Seleziona tutte le voci applicabili.

- Per scopi clinici
- Per scopi divulgativi (ad es. creare materiale informativo per pazienti)
- Per scopi scientifici (ad es. scrivere articoli scientifici)
- Altro: ____________________

22. Pensi che i sistemi di linguaggio generativo possano essere di ausilio:

Seleziona tutte le voci applicabili.

- Per aiutare i medici nella gestione dei pazienti
- Per aiutare i pazienti acquisire informazioni di natura medica
- Per aiutare i ricercatori della stesura di articoli scientifici

23. Quale è la tua percezione sui sistemi di linguaggio generativo (ad es. ChatGPT) correntemente disponibili?

1) Semplicità di utilizzo

Contrassegna solo un ovale.

	0	1	2	3	4	5	6	7	8	9	10	
Estr												Estremamente semplice

24. Quale è la tua percezione sui sistemi di linguaggio generativo (ad es. ChatGPT) correntemente disponibili?

2) Completezza delle informazioni generate

Contrassegna solo un ovale.

	0	1	2	3	4	5	6	7	8	9	10	
Per	◯	◯	◯	◯	◯	◯	◯	◯	◯	◯	◯	Estremamente complete

25. Quale è la tua percezione sui sistemi di linguaggio generativo (ad es. ChatGPT) correntemente disponibili?

3) Correttezza delle informazioni generate

Contrassegna solo un ovale.

	0	1	2	3	4	5	6	7	8	9	10	
Per	◯	◯	◯	◯	◯	◯	◯	◯	◯	◯	◯	Perfettamente corrette

26. Quale è la tua percezione sui sistemi di linguaggio generativo (ad es. ChatGPT) correntemente disponibili?

4) Comprensibilità delle informazioni generate

Contrassegna solo un ovale.

	0	1	2	3	4	5	6	7	8	9	10	
Per	◯	◯	◯	◯	◯	◯	◯	◯	◯	◯	◯	Perfettamente comprensibili

27. Utilizzi sistemi di intelligenza artificiale in altri ambiti della gastroenterologia? *

Contrassegna solo un ovale.

- Si
- No

28. Se si, quali?

Seleziona tutte le voci applicabili.

- [ ] Epatologia
- [ ] Pancreatologia
- [ ] IBD
- [ ] Fisiopatologia digestiva
- [ ] Oncologia gastrointestinale
- [ ] Altro: ______________________

SEZIONE 4 – Barriere e preoccupazioni

29. Quali pensi siano le principali barriere nella diffusione dell’intelligenza artificiale in gastroenterologia:

Seleziona tutte le voci applicabili.

- [ ] Costi
- [ ] Difficoltà di approvvigionamento da parte degli ospedali
- [ ] Assenza di conoscenza/consapevolezza da parte dei medici
- [ ] Assenza di linee guida sull’utilizzo

30. Quanto sei preoccupato nell'utilizzo dei sistemi di intelligenza artificiale in gastroenterologia?

1) Dalla affidabilità degli algoritmi dell'AI

Contrassegna solo un ovale.

	0	1	2	3	4	5	6	7	8	9	10	
Per	◯	◯	◯	◯	◯	◯	◯	◯	◯	◯	◯	Molto preoccupato

31. Quanto sei preoccupato nell'utilizzo dei sistemi di intelligenza artificiale in gastroenterologia?

2) Dal punto di vista legale

Contrassegna solo un ovale.

	0	1	2	3	4	5	6	7	8	9	10	
Per	◯	◯	◯	◯	◯	◯	◯	◯	◯	◯	◯	Molto preoccupato

32. Quanto sei preoccupato nell'utilizzo dei sistemi di intelligenza artificiale in gastroenterologia?

3) Dal punto di vista etico

Contrassegna solo un ovale.

	0	1	2	3	4	5	6	7	8	9	10	
Per	◯	◯	◯	◯	◯	◯	◯	◯	◯	◯	◯	Molto preoccupato

33. Quanto sei preoccupato nell'utilizzo dei sistemi di intelligenza artificiale in gastroenterologia?

4) Dal punto di vista regolatorio

Contrassegna solo un ovale.

	0	1	2	3	4	5	6	7	8	9	10	
Per	◯	◯	◯	◯	◯	◯	◯	◯	◯	◯	◯	Molto preoccupato

34. Quanto sei preoccupato nell'utilizzo dei sistemi di intelligenza artificiale in gastroenterologia?

5) Dalla sicurezza nella protezione dei dati

Contrassegna solo un ovale.

	0	1	2	3	4	5	6	7	8	9	10	
Per	◯	◯	◯	◯	◯	◯	◯	◯	◯	◯	◯	Molto preoccupato

SEZIONE 5 – Training e formazione

35. Pensi che l'AI debba essere utilizzata nel training dei giovani gastroenterologi? *

Contrassegna solo un ovale.

- ◯ Si, penso che possa incrementare e facilitare l'apprendimento e la formazione
- ◯ No, penso che possa costituire un handicap nei processi di apprendimento formazione
- ◯ Penso che sia ininfluente nell'influenzare l'apprendimento e la formazione

36. Quali pensi siano le forme educazionali più appropriate per formare i giovani gastroenterologi sull'AI?

Seleziona tutte le voci applicabili.

- [ ] Pratica clinica in sala
- [ ] Corsi hands-on
- [ ] Corsi online

SEZIONE 6 – Prospettive future

37. Come prevedi che l'integrazione dell'AI influenzerà la pratica endoscopica in futuro? *

Contrassegna solo un ovale.

- ( ) Impatto positivo
- ( ) Impatto negativo
- ( ) Impatto neutro

38. Sei ottimista riguardo al potenziale dell'AI nel migliorare le procedure endoscopiche? *

Contrassegna solo un ovale.

- ( ) Si
- ( ) No

39. Pensi che l'AI si integrerà facilmente nella pratica clinica? *

Contrassegna solo un ovale.

- ( ) Si
- ( ) No

40. Se si, entro quanti anni? *

____

41. Altri commenti

Questi contenuti non sono creati né avallati da Google.

Google Moduli

English version

Perception of artificial intelligence among Gastroenterologists in Italy: a national survey.

Dear colleague, we are conducting a national survey with the aim of evaluating the perception and acceptability of artificial intelligence methods among gastroenterologists in Italy.

We ask you to participate by completing an online questionnaire which requires approximately 5 minutes of your time. The answers provided will be treated anonymously and in aggregate form, solely for scientific purposes.

* Indicates a mandatory question

1. By continuing with this interview I give my consent to save and use the data provided for scientific and research purposes.

   Mark only one oval.

   ◯ I agree

   ◯ I do not agree (end of survey)

2. Are you a Consultant or Trainee in Gastroenterology ? *

Mark only one oval.

- Yes
- Not

SECTION 1 — Demographic Information

3. Name, Surname *

____

4. Age *

____

5. Genger *

Mark only one oval.

- Male
- Female
- Other

6. Specialization *

Mark only one oval.

- Consultant in Gastroenterology
- Trainee in Gastroenterology

7. Years of practice in Gastroenterology *

____

8. Hospital name *

9. Tipology of Hospital *

Mark only one oval.

- Universitary
- Not universitary
- Private

10. City *

11. Areas *

Mark only one oval.

- Northern
- Central
- Southern and Islands

SECTION 2 – Awareness and familiarity with AI

12. Have you heard about artificial intelligence in gastroenterology? *

Mark only one oval.

- Yes
- Not

13. How much do you know about the applications of artificial intelligence in gastroenterology?

Mark only one oval.

	0	1	2	3	4	5	6	7	8	9	10	
	◯	◯	◯	◯	◯	◯	◯	◯	◯	◯	◯	I consider myself an expert

SECTION 3 — Use of AI systems

14. Do you currently use artificial intelligence systems in endoscopy? *

Mark only one oval.

◯ Yes

◯ Not

15. If yes, which ones?

Check all that apply.

☐ Detection (CADe)

☐ Detection and characterisation (CADe/CADx)

☐ Endoscopic assessment of IBD

☐ AI systems for videocapsule endoscopy

☐ Other: ____________________

16. What is your perception of the currently commercialized AI endoscopy systems?

1) Ease of use

Mark only one oval.

	0	1	2	3	4	5	6	7	8	9	10	
	◯	◯	◯	◯	◯	◯	◯	◯	◯	◯	◯	Very easy

17. What is your perception of the currently commercialized AI endoscopy systems?

2) Diagnostic sensitivity

Mark only one oval.

	0	1	2	3	4	5	6	7	8	9	10	
s	◯	◯	◯	◯	◯	◯	◯	◯	◯	◯	◯	Risk of false negatives

18. What is your perception of the currently commercialized AI endoscopy systems?

3) Diagnostic specificity

Mark only one oval.

	0	1	2	3	4	5	6	7	8	9	10	
s	◯	◯	◯	◯	◯	◯	◯	◯	◯	◯	◯	Risk of false positives

19. What is your perception of the currently commercialized AI endoscopy systems?

4) Extension of the procedure times

Mark only one oval.

	0	1	2	3	4	5	6	7	8	9	10	
s	◯	◯	◯	◯	◯	◯	◯	◯	◯	◯	◯	High impact

20. Have you ever used generative language systems (e.g. ChatGPT) in medical practice?

Mark only one oval.

- ⬭ Yes
- ⬭ Not

21. If yes, for what purpose?

Check all that apply.

- ☐ For clinical purposes
- ☐ For outreach purposes (e.g. creating patient information materials)
- ☐ For scientific purposes (e.g. writing scientific papers)
- ☐ Other: ______________________

22. Do you think generative language systems can be helpful:

Check all that apply.

- ☐ To help clinicians manage patients
- ☐ To help patients acquire medical information
- ☐ To help researchers write scientific papers

23. What is your perception of currently available generative language systems (e.g. ChatGPT)?

1) Ease of use

Mark only one oval.

	0	1	2	3	4	5	6	7	8	9	10	
	⬭	⬭	⬭	⬭	⬭	⬭	⬭	⬭	⬭	⬭	⬭	Very easy

24. What is your perception of currently available generative language systems (e.g. ChatGPT)?

2) Completeness of generated information

Mark only one oval.

	0	1	2	3	4	5	6	7	8	9	10	
	◯	◯	◯	◯	◯	◯	◯	◯	◯	◯	◯	Extremely complete

25. What is your perception of currently available generative language systems (e.g. ChatGPT)?

3) Accuracy of generated information

Mark only one oval.

	0	1	2	3	4	5	6	7	8	9	10	
	◯	◯	◯	◯	◯	◯	◯	◯	◯	◯	◯	Perfectly correct

26. What is your perception of currently available generative language systems (e.g. ChatGPT)?

4) Understandability of generated information

Mark only one oval.

	0	1	2	3	4	5	6	7	8	9	10	
	◯	◯	◯	◯	◯	◯	◯	◯	◯	◯	◯	Perfectly understandable

27. Do you use AI systems in other areas of gastroenterology? *

Mark only one oval.

- Yes
- Not

28. If yes, which ones?

Check all that apply.

- [ ] Hepatology
- [ ] Pancreatology
- [ ] IBD
- [ ] Digestive physiopathology
- [ ] Gastrointestinal oncology
- [ ] Other: ______________________

SECTION 4 — Barriers and concerns

29. What do you think are the main barriers to the diffusion of artificial intelligence in gastroenterology:

Check all that apply.

- [ ] Costs
- [ ] Difficulty in supplying hospitals
- [ ] Lack of knowledge/awareness by clinicians
- [ ] Lack of guidelines on use

30. How concerned are you about using artificial intelligence systems in gastroenterology?

1) From the reliability of AI algorithms

Mark only one oval.

	0	1	2	3	4	5	6	7	8	9	10	
	◯	◯	◯	◯	◯	◯	◯	◯	◯	◯	◯	Very worried

31. How concerned are you about using artificial intelligence systems in gastroenterology?

2) From a legal point of view

Mark only one oval.

	0	1	2	3	4	5	6	7	8	9	10	
	◯	◯	◯	◯	◯	◯	◯	◯	◯	◯	◯	Very worried

32. How concerned are you about using artificial intelligence systems in gastroenterology?

2) From an ethical point of view

Mark only one oval.

	0	1	2	3	4	5	6	7	8	9	10	
	◯	◯	◯	◯	◯	◯	◯	◯	◯	◯	◯	Very worried

33. How concerned are you about using artificial intelligence systems in gastroenterology?

3) From a regulatory point of view

Mark only one oval.

	0	1	2	3	4	5	6	7	8	9	10	
	◯	◯	◯	◯	◯	◯	◯	◯	◯	◯	◯	Very worried

34. How concerned are you about using artificial intelligence systems in gastroenterology?

4) From security in data protection

Mark only one oval.

	0	1	2	3	4	5	6	7	8	9	10	
	◯	◯	◯	◯	◯	◯	◯	◯	◯	◯	◯	Very worried

SECTION 5 — Training and education

35. Do you think AI should be used in the training of young gastroenterologists? *

Mark only one oval.

- ◯ Yes, I think it can increase and facilitate learning and training.
- ◯ No, I think it could constitute a handicap in the learning and training processes.
- ◯ I think it is irrelevant in influencing learning and training.

36. What do you think are the most appropriate educational forms to train young gastroenterologists on AI?

Check all that apply.

- [ ] Clinical practice in the endoscopic room
- [ ] Hands-on courses
- [ ] Online courses

SECTION 6 – Future prospects

37. How do you foresee AI integration impacting endoscopic practice in the future? *

Mark only one oval.

- Positive impact
- Negative impact
- Neutral impact

38. Are you optimistic about the potential of AI to improve endoscopic procedures? *

Mark only one oval.

- Yes
- Not

39. Do you think AI will be easily integrated into clinical practice ? *

Mark only one oval.

- Yes
- Not

40. If yes, within how many years? *

____

41. Other comments

Google Moduli
