# Supplementary figures and images for: Perceptions of Artificial Intelligence Among Gastroenterologists in Italy: A National Survey
Source: Cancers (Basel). 2025 Apr 17;17(8):1353. doi: 10.3390/cancers17081353 (PMC12026144; doi:10.3390/cancers17081353)

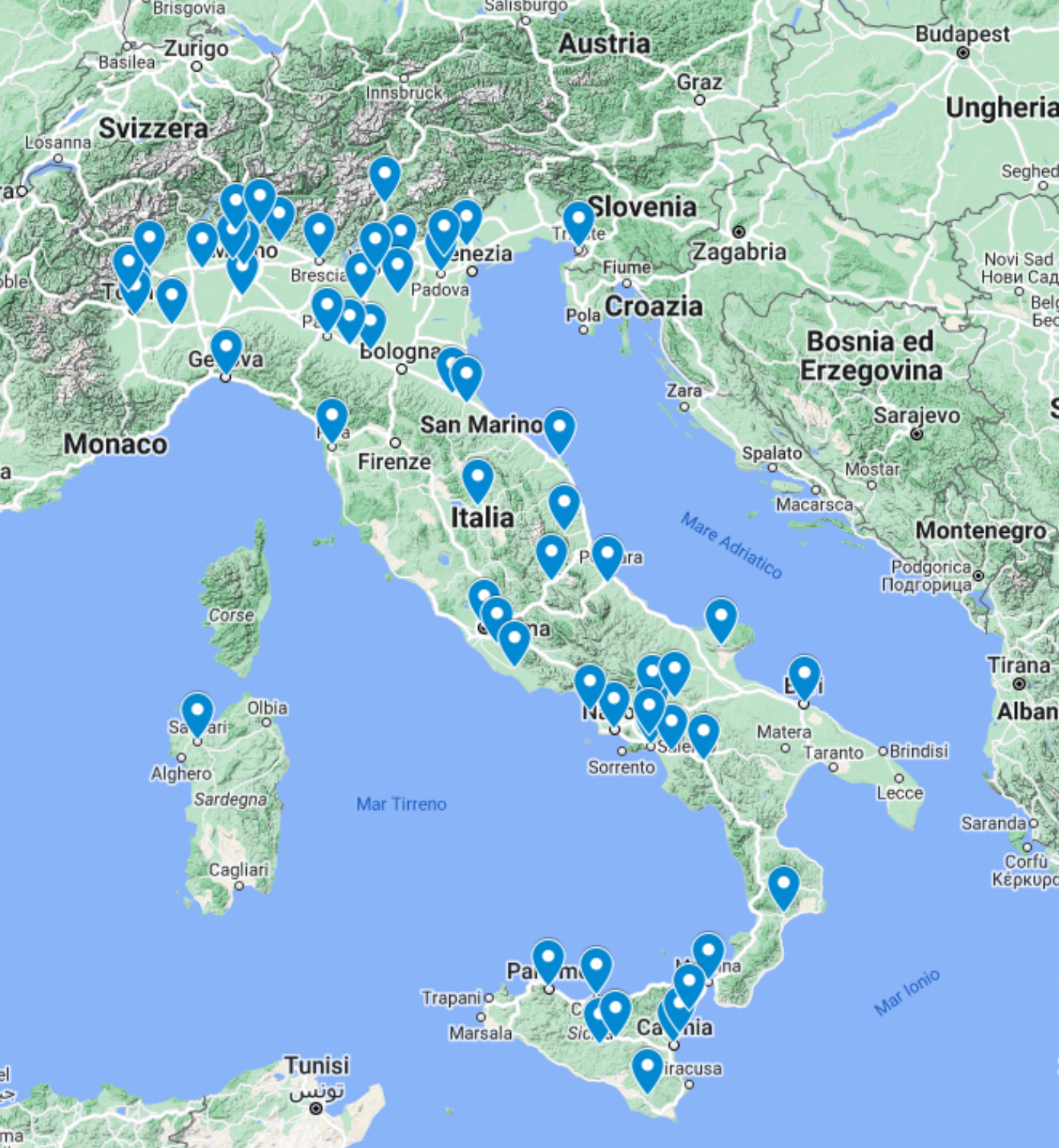

Supplement: Supplementary file 1 [file cancers-17-01353-s001.zip › Suppl Figure S1.pdf]
